# Adoptive Cell Therapy of Induced Regulatory T Cells Expanded by Tolerogenic Dendritic Cells on Murine Autoimmune Arthritis

**DOI:** 10.1155/2017/7573154

**Published:** 2017-06-18

**Authors:** Jie Yang, Lidong Liu, Yiming Yang, Ning Kong, Xueyu Jiang, Juan Sun, Rufeng Xie

**Affiliations:** ^1^Blood Engineering Laboratory, Shanghai Blood Center, Shanghai 200051, China; ^2^Division of Rheumatology, Huashan Hospital, Fudan University, Shanghai 200040, China

## Abstract

**Objective:**

Tolerogenic dendritic cells (tDCs) can expand TGF-*β*-induced regulatory T cells (iTregs); however, the therapeutic utility of these expanded iTregs in autoimmune diseases remains unknown. We sought to determine the properties of iTregs expanded by mature tolerogenic dendritic cells (iTreg_mtDC_) in vitro and explore their potential to ameliorate collagen-induced arthritis (CIA) in a mouse model.

**Methods:**

After induction by TGF-*β* and expansion by mature tDCs (mtDCs), the phenotype and proliferation of iTreg_mtDC_ were assessed by flow cytometry. The ability of iTregs and iTreg_mtDC_ to inhibit CD4^+^ T cell proliferation and suppress Th17 cell differentiation was compared. Following adoptive transfer of iTregs and iTreg_mtDC_ to mice with CIA, the clinical and histopathologic scores, serum levels of IFN-*γ*, TNF-*α*, IL-17, IL-6, IL-10, TGF-*β* and anti-CII antibodies, and the distribution of the CD4^+^ Th subset were assessed.

**Results:**

Compared with iTregs, iTreg_mtDC_ expressed higher levels of Foxp3 and suppressed CD4^+^ T cell proliferation and Th17 cell differentiation to a greater extent. In vivo, iTreg_mtDC_ reduced the severity and progression of CIA more significantly than iTregs, which was associated with a modulated inflammatory cytokine profile, reduced anti-CII IgG levels, and polarized Treg/Th17 balance.

**Conclusion:**

This study highlights the potential therapeutic utility of iTreg_mtDC_ in autoimmune arthritis and should facilitate the future design of iTreg immunotherapeutic strategies.

## 1. Introduction

Rheumatoid arthritis (RA) is an autoimmune disease causing chronic inflammation of the synovial joints. The inflammatory processes occurring in RA result in hyperplasia of the synovial membrane and infiltration of monocytes, macrophages, T and B cells, mast cells, and dendritic cells (DCs) [1]. Pharmacological therapies for RA include analgesics and anti-inflammatory steroids, which halt the progression of RA but do not cure it. Currently, a curative treatment has yet to be found. Therefore, the development of novel antirheumatic therapies that specifically target aberrant immune processes, dampen inflammation, and promote tolerance is needed.

Recently, cellular therapy for autoimmune diseases has attracted much attention, and as the master regulators of all immune responses, regulatory T cells (Tregs) are the most promising candidates for cell therapy. Natural Tregs (nTregs) are primarily derived from the thymus, and induced Tregs (iTregs) are differentiated from CD4^+^ CD25^−^ Foxp3^−^ T cells in the periphery or in vitro, both of which maintain immunological tolerance and may prevent a variety of autoimmune rheumatic diseases [2, 3]. According to previous reports, iTregs induced by TGF-*β* in vitro, but not nTregs, retain Foxp3 expression and immunosuppressive activity in the inflammatory microenvironment [4]. In addition, iTregs have been shown to suppress bone erosion and other clinical measures of disease progression in the well-established collagen-induced arthritis (CIA) mouse model of human RA [5, 6], suggesting that iTregs may be therapeutically beneficial for RA [7].

However, culturing iTregs for a period of 5 days has been reported to result in high levels of cell death (detected using propidium iodide staining) [8]. As shown in the study by Kong et al., 3 × 10^6^ iTregs per mouse (20 ± 2 g/mouse) were required to significantly inhibit established CIA [9]. The numbers of iTregs induced by TGF-*β* alone during conventional iTreg culture are not sufficient to satisfy therapeutic demands. Furthermore, after induction by TGF-*β*, only approximately 60% of CD4^+^ T cells express Foxp3 [8, 9], a percentage that is too low to meet the clinical requirements for relevant cell purity, although the remaining CD4^+^ Foxp3^−^ cells did not appear to have any pathogenic effects [10].

Fortunately, tolerogenic dendritic cells (tDCs), which typically present low levels of self-peptide-MHC complexes (signal 1) coupled with limited cell surface expression of costimulatory molecules (signal 2) and secretion of proinflammatory cytokines (signal 3), have been reported to potentially induce tolerance [11]. As the most potent antigen-presenting cells (APCs), DCs are regarded as key instigators or regulators of innate and adaptive immunity. Conventional DCs have the unique ability to activate or promote immune responses depending on their maturation status, whereas tDCs have the ability to induce activated T cell energy and apoptosis and generate/expand nTregs or iTregs in vivo or in vitro. Thus, tDCs are likely to act as both stimulators and inducers to further increase cell numbers and enhance Foxp3 expression in a mixed iTreg population [12].

In this study, we established a new polyclonal expansion method for the generation of iTregs. This method represents the first strategy for generating tDCs induced by IL-10/TGF-*β* in the Treg-induction/expansion system. Mature tDCs (mtDCs), which retained the tolerogenic functions of tDCs and had a stronger expansive ability than tDCs, were employed as the stimulator/inducer. We used mtDCs to successfully expand iTregs, while retaining their regulatory phenotype and potent suppressor functions. These mtDC-expanded iTregs (iTreg_mtDC_) were associated with a significant reduction in cytokine and CII-directed antibody secretion, polarization of the Treg/Th17 balance, and more effective inhibition of CIA than iTregs. Our findings suggest the potential use of iTreg_mtDC_ as a therapy for autoimmune arthritis.

## 2. Materials and Methods

### 2.1. Mice

Wild-type male DBA/1J (D1) mice (8 weeks old) were obtained from the Shanghai Laboratory Animal Center of the Chinese Academy of Science (SLACCAS, China). All mice were housed in a pathogen-free environment.

### 2.2. Ethics Statement

This study was conducted in strict accordance with the recommendations in the guidelines of the Institutional Animal Care and Use Committee of the Chinese Association for Laboratory Animal Sciences. The protocol was approved by the Committee on the Ethics of Animal Experiments of the Shanghai Blood Center (permit number: SBC-IRB-2013-07). All surgery was performed under diethyl ether anesthesia, and all efforts were made to minimize suffering.

### 2.3. Induction and Evaluation of CIA

CIA was induced in D1 mice via a subcutaneous injection of bovine type II collagen (CII, Chondrex, Redmond, WA, USA) emulsified with an equal volume of complete Freund's adjuvant (Difco, Detroit, MI, USA) on day 0. On day 21, mice received the next injection of 50 *μ*g of CII in incomplete Freund's adjuvant (Difco). The onset of CIA was confirmed on day 25. From day 21 to day 49 after the first immunization, mice were scored every two days for clinical evidence of arthritis of the limb joints by a macroscopic examination. Limb joint arthritis was scored using an established scoring system [13] as follows: no detectable arthritis, 0; erythema and mild swelling, 1; mild erythema and mild swelling involving the entire paw, 2; severe swelling and redness from the ankle to digits, 3; and maximal swelling and redness or obvious joint destruction associated with visible joint deformity or ankylosis, 4. The clinical scores for each mouse are presented as the sum of the scores for the four limbs, and the maximum score for each mouse was sixteen. Two independent observers without knowledge of the experimental protocol performed the scoring. During the 49-day observation period, the arthritis scores increased quickly and constantly and CIA developed in up to 90% of mice.

### 2.4. Preparation of DCs

DCs were prepared as described [13]. In brief, D1-derived bone marrow (BM) cells were collected and, after red cell lysis, seeded at a density of 5 × 10^5^ cells/ml in RPMI 1640 medium supplemented with 10% fetal bovine serum (Invitrogen). On day 0, murine GM-CSF (20 ng/ml, PeproTech) was added to the cultures, following which the cultures were pulsed with fresh medium, GM-CSF, murine IL-10 (15 ng/ml, PeproTech), and human TGF-*β*1 (15 ng/ml, PeproTech) on days 4 and 7. After 10 days in culture, nonadherent cells were collected and CD11c^+^ cells were sorted as the tDCs. mtDCs were harvested after stimulation with 100 ng/ml lipopolysaccharide (LPS, Sigma-Aldrich) during the final 48 h of culture. Immature DCs (iDCs) were propagated in the presence of GM-CSF alone under the same conditions, and mature DCs (mDCs) were generated following exposure to LPS for 48 h.

All types of DCs were stained with anti-mouse CD11c-APC, CD86-FITC, CD80-PE, and IA-IE-PE mAbs (BD Biosciences Pharmingen) for phenotypic analysis by flow cytometry. Cytokine expression was determined using real-time quantitative PCR. Primer sequences (in the 5′ to 3′ orientation) were IL-12 p40, GGAAGCACGGCAGCAGAATA and AACTTGAGGGAGAAGTAGGAATGG; TGF-*β*, TTGCTTCAGCTCCACAGAGA and TGGTTGTAGAGGGCAAGGAC; IL-10, CCAAGCCTTATCGGAAATGA and TTTTCACAGGGGAGAAATCG; and *β*-actin, ATCCGTAAAGACCTCTATGC and ACACAGAGTACTTGCGCTCA. PCR results were normalized to the expression of the housekeeping gene *β*-actin [14]. Data shown are a representative of six independent experiments.

### 2.5. Ex Vivo Generation of iTreg_mtDC_

iTregs were derived from CD4^+^ CD25^−^ T cells that were purified from the splenocytes of D1 mice using a CD4^+^ CD25^+^ regulatory T cell isolation kit (Miltenyi Biotec, Germany) and stimulated with an anti-CD3/CD28 mAb (50 ng/ml, PeproTech) in the presence of TGF-*β*1 (5 ng/ml, PeproTech) and IL-2 (100 ng/ml, PeproTech) for 5 days [8]. Then, iTreg_mtDC_ or iTreg_mDC_ were generated by expanding iTregs for 4 days using mtDCs or mDCs at a 5 : 1 ratio (5 T cells : 1 DC). The different types of iTregs were collected, counted, incubated with anti-mouse CD4-FITC and CD25-PE-Cy5 mAbs, fixed, permeabilized, and stained with antimouse Foxp3-PE or isotype control mAbs (BD Biosciences Pharmingen). Then, the coexpression of CD25 and Foxp3 was determined by flow cytometry.

### 2.6. Proliferation Assay

In the classical polyclonal proliferation assay, responder cells were CD4^+^ T cells, which were isolated from splenocytes of D1 mice using a CD4^+^ T Cell Isolation Kit (Miltenyi Biotec, Germany). The CD4^+^ T cells were labeled with carboxyfluorescein succinimidyl ester (CFSE; Invitrogen, Germany) and stimulated with anti-CD3/28 mAbs (50 ng/ml, PeproTech). Then, different ratios of iTregs, iTreg_mDC_, or iTreg_mtDC_ (as suppressor cells) were added to the responder cultures. Four days later, the cells were harvested and analyzed by flow cytometry.

In addition, CD4^+^ T cells were isolated from splenocytes of mice with CIA (on day 25 after the primary immunization) and used in the antigen-specific CD4^+^ T cell proliferation assay. Mature DCs were induced as described above and loaded with CII, as stimulator APCs. CFSE-labeled responding CD4^+^ T cells were activated following coculture with CII-loaded mDCs. Then, different ratios of iTregs, iTreg_mDC_, or iTreg_mtDC_ (as suppressor cells) were added to the responder cultures. Four days later, the cells were harvested and analyzed using flow cytometry.

### 2.7. Evaluation of CIA after iTreg_mtDC_ Treatment

At the onset of CIA, 1 × 10^6^ iTregs, iTreg_mDC_, or iTreg_mtDC_ were adoptively transferred into CIA-recipient mice (*n* = 10 per group) via the tail vein. Control mice were treated with PBS alone. Mice were scored using an established scoring system from days 21 to 49 after the primary immunization [13].

### 2.8. Histology

The hind paws of iTreg-treated, iTreg_mtDC_-treated, and CIA mice were collected on day 49 after the primary immunization, and the tissues were stained with hematoxylin and eosin (H&E) and Safranin O. Two independent observers who were blinded to the experimental groups examined the paw sections using a four-point scale: normal, 0; inflammatory infiltrates and synovial hyperplasia, 1; pannus formation and cartilage erosion, 2; and import cartilage erosion and bone destruction, 3. This global histological score reflected both synovitis (synovial proliferation and inflammatory cell infiltration) and joint destruction (bone and cartilage thickness, irregularity, and the presence of erosions) [13].

### 2.9. Analysis of Serum Anti-CII Antibody and Cytokine Levels Using CBA and ELISA

Sera were obtained from iTreg-treated, iTreg_mtDC_-treated, and CIA mice 49 days after the primary immunization and stored at −80°C. The serum levels of IFN-*γ*, TNF-*α*, IL-17, IL-6, and IL-10 were determined using a mouse CBA Kit (BD Biosciences Pharmingen) and analyzed by flow cytometry. The concentration of TGF-*β* was assessed using the mouse TGF-*β* Platinum ELISA kit (eBioscience), and the concentrations of anti-CII antibodies were measured using a standard sandwich ELISA (Chondrex, Redmond, WA, USA) according to the manufacturer's instructions. Five samples from each group were analyzed.

### 2.10. Analysis of Treg/Th17 Subsets in Mice with CIA

T cells were isolated from the spleen and inguinal lymph nodes of the iTreg-treated, iTreg_mtDC_-treated, and CIA mice on day 49 after the primary immunization. These cells were incubated with an anti-mouse CD4-FITC mAb, fixed, permeabilized, stained with anti-mouse FoxP3-PE, IL-17-PE, or isotype control mAbs (BD Biosciences Pharmingen), and then assessed by flow cytometry.

### 2.11. Th17 Cell Differentiation

Naive CD4^+^ T cells were sorted from the splenic cells of mice with CIA, stained with CFSE as described above, and stimulated with an anti-CD3/CD28 mAb (50 ng/ml, PeproTech), IL-1*β* (10 ng/ml, PeproTech), IL-6 (25 ng/ml, PeproTech), and TGF-*β*1 (1 ng/ml, PeproTech). Then, iTregs, iTreg_mDC_, or iTreg_mtDC_ were added to the culture at a 1 : 1 ratio. After 4 days of coculture, the cells were harvested, incubated with an anti-mouse CD4-APC mAb, fixed, permeabilized, and stained with an anti-IL-17A-PE mAb (BD Biosciences Pharmingen). The production of IL-17A in the cell culture supernatant was measured using the mouse cytokine cytometric bead array (CBA, BD Biosciences Pharmingen) according to the manufacturer's instructions.

### 2.12. Statistical Analyses

Results were analyzed using GraphPad Prism 5.0 software (GraphPad Software, San Diego, CA, USA), and data are expressed as means ± standard errors of the means (SEM). Student's *t*-test was used to assess statistically significant differences between two paired groups, and an alpha value of *P* < 0.05 was considered statistically significant.

## 3. Results

### 3.1. BM-Derived DCs Induced by IL-10 and TGF-*β*1 Maintain the Tolerogenic Surface Phenotype and Express Substantial Levels of “Immunosuppressive” Cytokines

**Figure 1:**
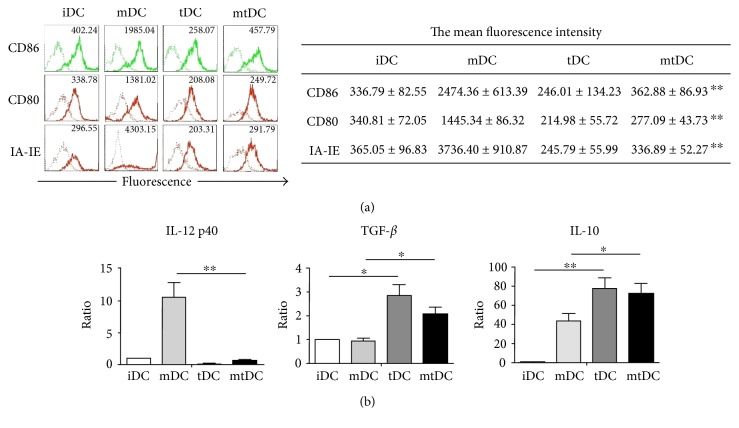
Characteristic profile of tolerogenic DCs derived from D1 mice.iDCs, mDCs, tDCs, and mtDCs were induced to differentiate in vitro, as described in the Materials and Methods, after which CD11c^+^ cells were harvested. (a) These four types of DC were stained with CD86, CD80, IA-IE (thick lines), or isotype-matched mAbs (thin lines), and the expression of these markers was analyzed by FACS using flow cytometry. The frequency of positively stained cells and MFI of a representative of 10 independent experiments are shown in the FACS profile. The mean fluorescence intensity is reported as the mean ± SEM (*n* = 10). ^∗∗^*P* < 0.01 for the comparison of mDCs with mtDCs using unpaired *t*-tests. (b) IL-12 p40, IL-10, and TGF-*β* expression in DCs was determined by real-time PCR. All results were normalized to the expression of the housekeeping gene *β*-actin and are expressed as the means ± SEM of six independent experiments. ^∗^*P* < 0.05 and ^∗∗^*P* < 0.01 for the comparisons of the indicated groups using the unpaired *t*-test.

According to previous reports, tDCs derived from mouse BM are induced to differentiate with GM-CSF, IL-10, and TGF-*β*1, which does not affect DC development from replicating BM progenitors [15]. In the present study, a large proportion of induced BM cells were positive for CD11c (>80%), indicating that these cells exhibited a DC phenotype. MHC molecules (IA-IE) and costimulatory molecules (CD86 and CD80) were expressed at low levels on tDCs. Following stimulation with LPS for 48 h, the mean fluorescence intensity (MFI) of each of these molecules was consistently lower on the mature tDCs (mtDCs) than on the mature DCs (mDCs) (Figure 1(a)). Thus, tDCs were comparatively resistant to maturation in response to LPS stimulation and retained the tolerogenic surface phenotype. Additionally, cytokine production by DCs was assessed using real-time PCR. The tDCs displayed higher levels of “immunosuppressive” cytokines, such as IL-10 and TGF-*β*, than iDCs. These cytokines were still expressed at high levels after tDCs were stimulated with LPS, although the expression of the TGF-*β* mRNA in mtDCs was lower than, but not significantly different from, that in tDCs. However, IL-12 p40 production by tDCs was negligible, even upon stimulation with LPS (Figure 1(b)). There was no significant difference on induction efficiency and cell yields between iDCs, mDCs, tDCs, and mtDCs, though the frequency of CD11c^+^ cells were increased after LPS stimulation.

Based on these data, tDCs induced by GM-CSF, IL-10, and TGF-*β*1 maintained a tolerogenic surface phenotype, even after LPS stimulation, and expressed substantial levels of “immunosuppressive” cytokines.

### 3.2. iTregs Generated after mtDC-Mediated Expansion Retain Foxp3 Expression and Potently Suppress Polyclonal CD4^+^ T Cell Proliferation In Vitro

**Figure 2:**
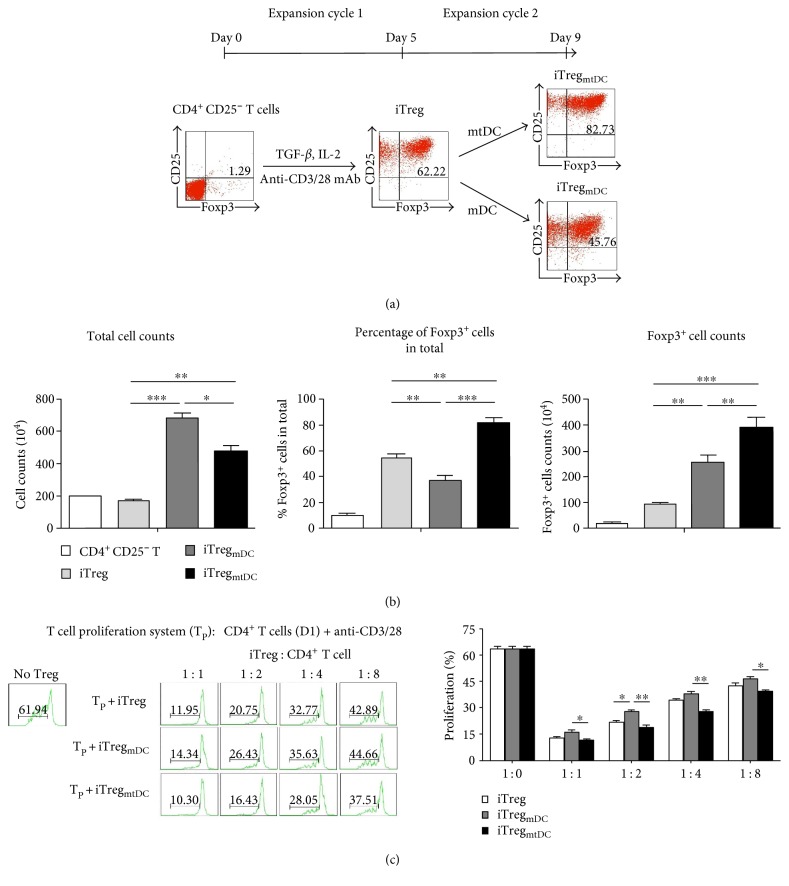
iTregs expanded in vitro by mtDCs retained Foxp3 expression and efficiently inhibited CD4^+^ T cell proliferation. (a) Schematic overview of the induction/expansion strategy for iTregs. The two expansion cycles were established as described in the Materials and Methods. The differentiation of iTregs was first induced with an anti-CD3/CD28 mAb, TGF-*β*, and IL-2 for 5 days in vitro. Then, iTreg_mtDC_ or iTreg_mDC_ were generated by expanding iTregs for 4 days using mtDCs or mDCs, respectively. The expression of the transcription factor Foxp3 and CD25 in cells was determined in each cycle using flow cytometry. Data are representative of three independent experiments. (b) Expansion of different types of iTregs was determined by counting the cells, and Foxp3 expression levels were determined by flow cytometry. All data are presented as means ± SEM (*n* = 5), and ^∗^*P* < 0.05, ^∗∗^*P* < 0.01, and ^∗∗∗^*P* < 0.001 for the comparisons of the indicated groups using the unpaired *t*-test. (c) The ability of iTregs to inhibit the proliferation of CD4^+^ T cells was assessed using the proliferation assay. In this assay, CD4^+^ T cells (responders) were isolated from splenocytes of D1 mice, stained with CFSE, and stimulated with an anti-CD3/CD28 mAb and IL-2. Then, different ratios of iTregs, iTreg_mDC_, or iTreg_mtDC_ (suppressor cells) were added to the responder cultures. Four days later, the cells were harvested and analyzed by flow cytometry. Progressive dilution of CFSE in responder cells was used as a readout of proliferation in the presence or absence of different types of iTregs. The results are presented as means ± SEM (*n* = 3). ^∗^*P* < 0.05 and ^∗∗^*P* < 0.01 compared with the indicated groups using the unpaired *t*-test.

Our iTreg culture strategy consisted of two expansion cycles: (1) the induction of iTregs and (2) the expansion of iTregs by mtDCs (Figure 2(a)). First, CD4^+^ CD25^−^ T cells that were purified from splenocytes of D1 mice were stimulated with an anti-CD3/CD28 mAb (50 ng/ml, PeproTech) in the presence of TGF-*β*1. After 5 days, these cells, “iTregs,” were harvested. Then, iTregs were cocultured with mtDCs at a 5 : 1 ratio. After 4 days, the cells, termed “iTreg_mtDC_,” were collected, and a significant increase in the total cell number was observed. According to the FACS results, the intracellular expression of Foxp3 in iTreg_mtDC_ was markedly higher (82.0 ± 3.6%) than that in iTregs (54.9 ± 2.5%). Using our new culture method, we obtained 3.52–4.3 × 10^6^ Foxp3^+^ cells from the 2 × 10^6^ CD4^+^ CD25^−^ T cells that were originally plated and included approximately 0.02 × 10^6^ Foxp3^+^ cells (Figure 2(b)). In other words, the Foxp3^+^ cells had expanded approximately 150-fold compared with original cells. However, the iTreg_mDC_, which were expanded by mDCs, appeared to expand more rapidly but generated a lower percentage of cells that expressed Foxp3 (37.4 ± 4.1%) than the iTreg_mtDC_.

iTregs exhibit immunosuppressive activity. Therefore, the classical polyclonal effector T cell proliferation assay was used as a functional readout to show the inhibitory capacity of iTregs expanded by mtDCs. In this assay, CD4^+^ T cells (responders) were isolated from splenocytes of D1 mice and stimulated with anti-CD3/28 mAbs. As shown in Figure 2(c), the responder cells underwent vigorous proliferation in the absence of suppressors, generating large numbers of dividing T cells in culture. However, this vigorous proliferation was suppressed by the presence of iTregs, iTreg_mtDC_, or iTreg_mDC_ (suppressors). Significantly, iTreg_mtDC_ inhibited CD4^+^ T cell proliferation more effectively than iTregs or iTreg_mDC_ at all the tested suppressor-to-responder (S : R) cell ratios. Even the lowest dose of iTreg_mtDC_ (an S : R ratio of 1 : 8) resulted in the effective inhibition of CD4^+^ T cell expansion, whereas both iTregs and iTreg_mDC_ showed weaker suppression at the same S : R ratio.

Based on these results, iTregs were expanded by mtDCs and maintained the stronger regulatory phenotype and more effective inhibitory potency.

### 3.3. In CIA Mice, iTreg_mtDC_ Exhibited More Potent Antiarthritic Activity than iTregs

As shown in previous studies, iTregs exert protective effects on established arthritis [4, 9]; therefore, we determined the capacity of iTreg_mtDC_ to improve established CIA. Mice with CIA were injected with the same dose (1 × 10^6^ cells/animal) of either iTregs, iTreg_mDC_, or iTreg_mtDC_, and then, the arthritic index and histopathology were examined to assess the antiarthritic activities of these three types of iTregs.

**Figure 3:**
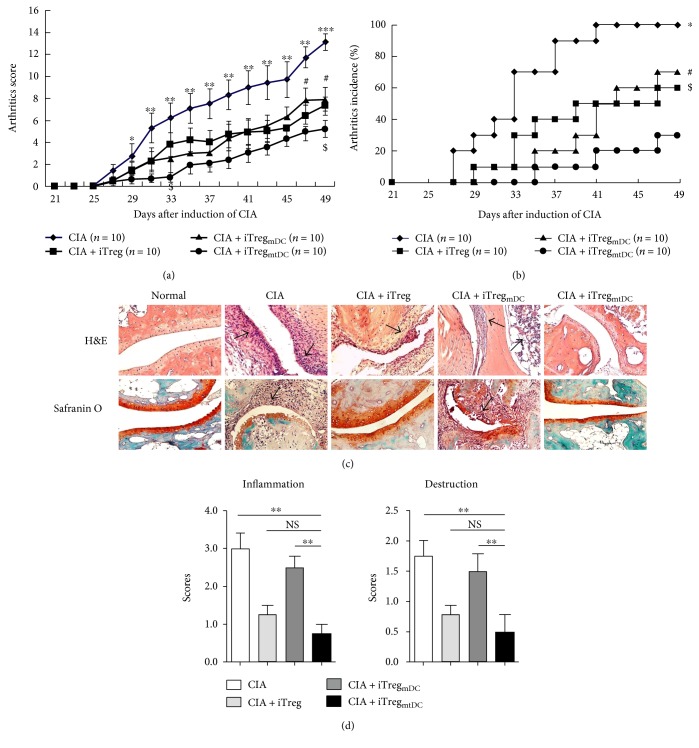
iTreg_mtDC_ exhibited a more potent antiarthritic activity in CIA mice than iTregs. Following the onset of experimentally induced CIA (on day 25 after the primary immunization), recipient mice received adoptively transferred iTregs, iTreg_mDC_, or iTreg_mtDC_ (1 × 10^6^ cells/animal). Mice were scored for clinical signs of arthritis in the limb joints by macroscopic examination three times per week. Limb joint arthritis was assessed using an established scoring system. The mice were sacrificed on the 49th day after the first immunization with CII. (a) Arthritic scores and incidence for each group following the adoptive transfer of different types of iTregs (*n* = 10) during the observation period are shown. ^∗^*P* < 0.05, ^∗∗^*P* < 0.01, and ^∗∗∗^*P* < 0.001 for the comparison of the CIA group and the CIA + iTreg_mtDC_ group; ^#^*P* < 0.05 for the comparison of the CIA + iTreg group and the CIA + iTreg_mtDC_ group; and ^$^*P* < 0.05 for the comparison of the CIA + iTreg_mDC_ group and the CIA + iTreg_mtDC_ group using the unpaired *t*-test. (b) Hind paw specimens were collected from recipient mice treated with different types of iTregs on the 49th day after the first immunization with CII and were stained with H&E (top, synovial joint inflammation) and Safranin O (bottom, cartilage erosion). All specimens were shown at ×200. (c) Histopathological scores for inflammation and destruction in each group are expressed as the means ± SEM of four individual experiments. ^∗∗^*P* < 0.01 for the comparisons of the indicated groups using the unpaired *t*-test.

According to the arthritic scores, all types of iTregs markedly decreased the incidence of CIA and reduced the severity of arthritis compared with that of the untreated control group. More importantly, the injection of iTreg_mtDC_ almost completely inhibited the progression of arthritis during the first 10 days, and subsequently, the arthritic severity never reached the level observed in the CIA control group. When compared with the therapeutic effects of iTregs and iTreg_mDC_ at any time point, treatment with the same doses of iTreg_mtDC_ inhibited the development of CIA to a greater extent, which was substantiated by both the clinical scores and the incidence of arthritis (Figures 3(a) and 3(b)). During the 4-week observation period, iTreg_mtDC_-treated mice had the mildest arthritic symptoms and the lowest arthritis scores compared with those mice treated with iTregs or iTreg_mDC_.

Next, histopathological specimens of the joints of mice with CIA treated with iTreg_mtDC_ showed the least significant cartilage destruction and least inflammatory cell infiltration compared with those of control CIA mice, although the iTreg or iTreg_mDC_ treatment also reduced the joint damage and inflammation to some extent (Figure 3(c)). The results of the histological examinations were consistent with the resulting clinical scores (Figure 3(d)).

Thus, these in vivo observations confirmed that treatment of CIA mice with iTreg_mtDC_ inhibits the progression of CIA more effectively than the iTreg or iTreg_mDC_ treatment.

### 3.4. iTreg_mtDC_-Mediated Inhibition of CIA Was Associated with Modulated Cytokine and Anti-CII Antibody Secretion and the Polarization of the Treg/Th17 Balance

The amelioration of CIA in mice following the iTreg treatment has been attributed to the immune-modulating properties of iTregs [4]. Therefore, we sought to characterize the mechanism by which iTreg_mtDC_ modulates the disease.

**Figure 4:**
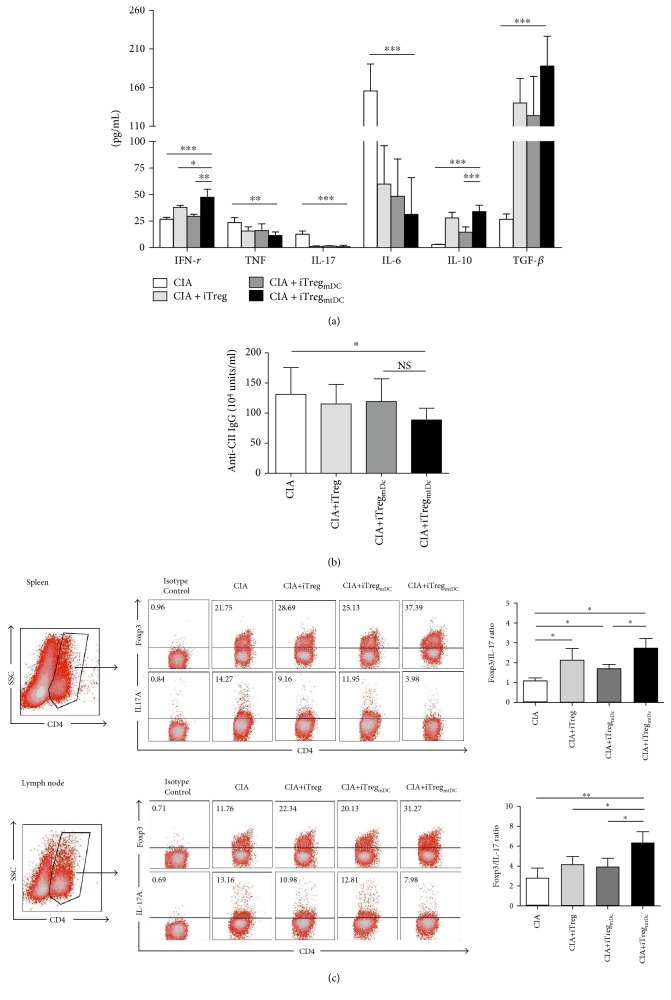
In the CIA mouse model, iTreg_mtDC_ modulated the secretion of cytokines and anti-CII antibodies and polarized the Treg/Th17 balance. Recipient mice that received adoptively transferred iTregs, iTreg_mDC_, or iTreg_mtDC_ were sacrificed on the 49th day after the first immunization with CII, and serum was collected from each group of mice. (a) Serum levels of IFN-*γ*, TNF-*α*, IL-17, IL-6, and IL-10 were measured using CBA assays, and TGF-*β* secretion was measured using an ELISA. Data are reported as means ± SEM (*n* = 5). ^∗^*P* < 0.05, ^∗∗^*P* < 0.01, and ^∗∗∗^*P* < 0.001 for the comparisons of the indicated groups using the unpaired *t*-test. (b) Serum levels of total CII-specific immunoglobulin were determined using an ELISA. The results from five independent replicate experiments were pooled. Data are reported as means ± SEM (*n* = 5). ^∗^*P* < 0.05 and ^∗∗^*P* < 0.01 for the comparisons of the indicated groups using the unpaired *t*-test. (c) CD4^+^ T cells from the spleen and inguinal lymph nodes of CIA mice treated with or without different types of iTregs were collected, permeabilized, and stained with an anti-Foxp3 mAb and an anti-IL-17 mAb to detect intracellular expression. FACS flow cytometry was used to measure the percentage of positively stained cells, and the ratios of Treg/Th17 cells are expressed as the means ± SEM of five independent experiments. ^∗^*P* < 0.05 and ^∗∗^*P* < 0.01 for the comparisons of the indicated groups using the unpaired *t*-test.

First, the secretion of IFN-*γ*, TNF-*α*, IL-17, IL-6, IL-10, and TGF-*β* in the sera of CIA mice treated with various iTregs was analyzed on day 49 after the first immunization. The production of IFN-*γ* and IL-10 significantly increased in iTreg_mtDC_-treated mice compared with those productions in iTreg- or iTreg_mDC_-treated mice. In addition, the levels of TNF, IL-17, and IL-6 were significantly reduced and TGF-*β* was markedly elevated in iTreg-treated, iTreg_mDC_-treated, or iTreg_mtDC_-treated mice compared with CIA mice, though there were no significant differences on the levels of those cytokines between the groups treated with different iTregs (Figure 4(a)). Clearly, the attenuation of CIA in mice following adoptive transfer of iTregs was a consequence of their immune modulatory effect on the secretion of various cytokines, and iTreg_mtDC_ showed the strongest modulating effect.

Additionally, the importance of antibodies in the development of CIA pathology is also well described [16]. The serum levels of total anti-CII-specific immunoglobulin (anti-CII IgG) in iTreg_mtDC_-treated mice were significantly lower than the levels observed in mice with CIA on day 49 following the first immunization, whereas treatment with either iTregs or iTreg_mDC_ failed to obviously reduce anti-CII IgG levels. And there was no significant difference on the levels of anti-CII IgG between iTreg_mtDC_-treated mice and iTregs- or iTreg_mDC_-treated mice (Figure 4(b)). Thus, iTreg_mtDC_ inhibited anti-CII-specific antibody responses more powerfully than iTregs or iTreg_mDC_, which contributed to the suppression of the development of CIA.

Furthermore, the Treg/Th17 ratio is considered an important indicator of the severity of CIA. Thus, we determined the percentages of Th17 (CD4^+^ IL-17^+^) and Treg (CD4^+^ Foxp3^+^) cells among CD4^+^ T cells isolated from the spleen or inguinal lymph nodes of CIA mice that were treated with various iTregs on day 49 after the first immunization. As shown in Figure 4(c), the Foxp3 : IL-17 ratio increased due to an increase in the frequency of Treg cells and a decrease in the frequency of Th17 cells following all types of iTreg treatment. Obviously, the highest Foxp3 : IL-17 ratio was detected in iTreg_mtDC_-treated mice compared with that in either iTreg- or iTreg_mDC_-treated mice. Thus, iTreg_mtDC_ exerted the most significant modulatory effects on the polarization of the Treg/Th17 balance in vivo.

Taken together, compared to iTregs and iTreg_mDC_, the adoptive transfer of iTreg_mtDC_ exerted the most obvious suppression of cytokine secretion, reduction of CII-directed antibodies, and the most significant polarization of the Treg/Th17 balance.

### 3.5. iTreg_mtDC_ Inhibit the Antigen-Specific Proliferation of CIA-CD4^+^ T Cells and Suppress Th17 Cell Differentiation In Vitro More Effectively than iTregs

**Figure 5:**
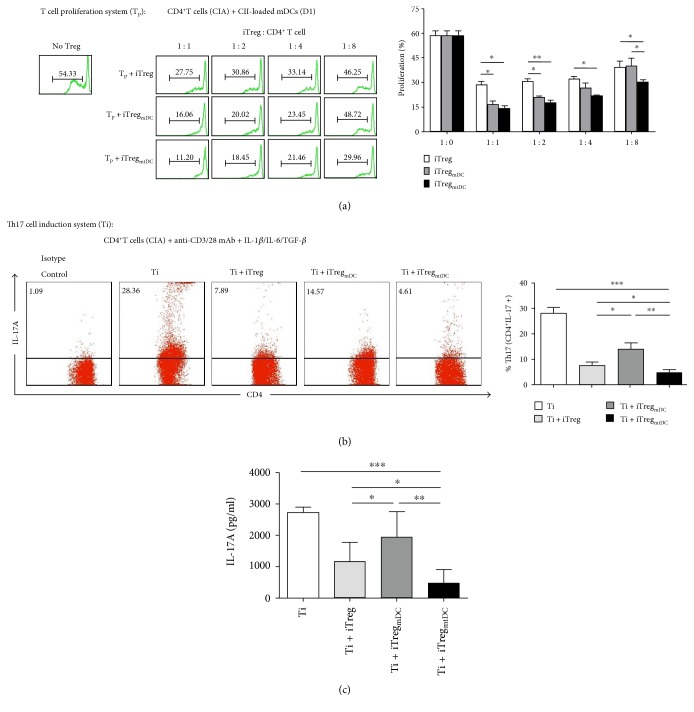
iTreg_mtDC_ exerted a more potent immunosuppressive effect on antigen-specific CD4^+^ T cell proliferation and suppressed Th17 cell differentiation more effectively. (a) The ability of iTreg_mtDC_ to inhibit the proliferation of antigen-specific CD4^+^ T cells was assessed using the proliferation assay. In this assay, CD4^+^ T cells (responders) were isolated from splenocytes of CIA mice (on day 25 after the primary immunization), stained with CFSE, and stimulated with CII-loaded mDCs (stimulators) derived from the BM of normal D1 mice. Then, different ratios of iTregs, iTreg_mDC_, or iTreg_mtDC_ (suppressor cells) were added to the responder cultures. Four days later, the cells were harvested and analyzed by flow cytometry. The results are shown as means ± SEM (*n* = 3). ^∗^*P* < 0.05 and ^∗∗^*P* < 0.01 for the comparisons of the indicated groups using the unpaired *t*-test. (b) The suppression of Th17 cell differentiation by coculture with iTreg_mtDC_ was assessed using the standard Th17 cell differentiation system. As described in the Materials and Methods, naive CD4^+^ T cells were isolated from the splenocytes of CIA mice, stained with CFSE, and stimulated with an anti-CD3/CD28 mAb, IL-1*β*, IL-6, and TGF-*β*1. Then, a 1 : 1 ratio of iTregs, iTreg_mDC_, or iTreg_mtDC_ was added to this differentiation system. After 4 days of cocultivation, the percentages of CFSE^+^ CD4^+^ IL-17A^+^ cells in the different groups were determined by flow cytometry as the percentage of induced Th17 cells. Data are reported as means ± SEM (*n* = 3), and ^∗^*P* < 0.05, ^∗∗^*P* < 0.01, and ^∗∗∗^*P* < 0.001 represent the comparisons of the indicated groups using unpaired *t*-test. (c) After 4 days of cocultivation, supernatants were collected from the groups mentioned in (b). The concentration of soluble IL-17A in the coculture supernatant was determined using CBA. Data are reported as means ± SEM (*n* = 3), and ^∗^*P* < 0.05, ^∗∗^*P* < 0.01, and ^∗∗∗^*P* < 0.001 represent the comparisons of the indicated groups using the unpaired *t*-test.

We utilized an in vitro antigen-specific proliferation assay to simulate the in vivo response as a functional readout and to demonstrate the ability of iTreg_mtDC_ to suppress the antigen-specific proliferation of CIA-CD4^+^ T cells. In this assay, CD4^+^ T cells (responders) were isolated from splenocytes of CIA mice and stimulated with CII-loaded mDCs (stimulators). As shown in Figure 5(a), a large number of CIA-CD4^+^ T cells underwent antigen-specific amplification after expansion by CII-loaded mDCs. However, no matter if iTregs, iTreg_mtDC_, or iTreg_mDC_ (suppressors) were added to the proliferation assay, this strong proliferation was obviously inhibited. At all tested S : R ratios, iTreg_mtDC_ inhibited antigen-specific CD4^+^ T cell expansion more effectively than iTregs. Notably, a significant difference between the abilities of iTreg_mtDC_ and iTreg_mDC_ to inhibit the antigen-specific CD4^+^ T cell expansion was only observed when they were added at a low dose (at an S : R ratio of 1 : 8); iTregs showed weaker suppression at the same S : R ratio.

Given the close relationship between Tregs and Th17 cells [17] and our observations that the adoptive transfer of iTregs decreased the number of Th17 cells and subsequent polarization of the Treg/Th17 balance in the CIA mice, we next investigated whether iTreg_mtDC_ could maintain suppression of Th17 differentiation in vitro, which simulated the in vivo response. In the Th17 induction system, Th17 cells were differentiated from CIA-CD4^+^ T cells by stimulation with IL-6, IL-1*β*, and TGF-*β* for 4 days. The 1 : 1 ratio of iTreg_mtDC_ to CIA-CD4^+^ T cells markedly reduced the fraction of cells that expressed intracellular IL-17A from 28.12 ± 4.79% to 4.83 ± 1.53%. The addition of the same ratio of iTregs and iTreg_mDC_ to CD4^+^ T cells achieved a weaker suppression of intracellular IL-17A expression (Figure 5(b)). Meanwhile, the level of soluble IL-17A, the key cytokine which Th17 secreted, detected in the supernatant also decreased significantly upon the addition of iTreg_mtDC_ (Figure 5(c)).

Based on these results, iTreg_mtDC_ were the most effective at inhibiting antigen-specific CIA-CD4^+^ T cell proliferation, suppressed Th17 cell differentiation to a greater extent and decreased the secretion of IL-17 in vitro compared with iTregs or iTreg_mDC_, providing strong evidence that adoptive transfer of iTreg_mtDC_ reflected more potent antiarthritic activity in the CIA mice.

## 4. Discussion

CD4^+^ CD25^+^ Foxp3^+^ regulatory T cells play a crucial role in maintaining immune tolerance. As shown in multiple studies, nTregs prevent the appearance and development of autoimmune diseases in many animal models [18, 19]. However, nTregs are unable to treat established autoimmune diseases because of their instability in an inflammatory milieu [20–22]. In addition, the widespread use of nTreg-based therapy is hindered by the low frequency and lack of reliable surface markers for purification [23, 24]. In 2002, Zheng et al. were the first to report that TGF-*β* induces the differentiation of CD4^+^ CD25^−^ cells into CD4^+^ CD25^+^ Tregs (iTregs) in vitro [25]. Over the past decade, a number of studies on iTregs and their relationship to the regulation of immunity and the outcomes of autoimmune diseases have shown that adoptive transfer of Foxp3^+^ iTregs ameliorates autoimmune disease in mouse models of lupus, gastritis, diabetes, and RA [4, 26–28], although the capacity to apply this therapy in the clinic remains controversial [29, 30]. In this study, we established a new method for the polyclonal expansion of iTregs using mtDCs and assessed the suppressive capacity of iTreg_mtDC_ in vitro and in CIA mice. According to our results, iTreg_mtDC_ corrected defects in the number and function of iTregs in vivo and in vitro and thus possessed the potential to be a powerful tool in the treatment of autoimmune diseases.

In the classical iTreg culture method, iTreg differentiation is induced by an anti-CD3/CD28 antibody and TGF-*β*. This method produces few cells due to a high cell death rate, and the number of cells obtained is not sufficient to meet therapeutic demands. We modified the iTreg-induction/culture protocol to produce more cells and to promote the clinical application of iTreg-based therapy. In the conventional polyclonal expansion method, an anti-CD3/28 antibody or mature DCs are used as stimulators, and the addition of high doses of anti-CD3/28 antibody led to severe cell apoptosis caused by excessive activation, as the anti-CD3/28 antibody preferentially amplified CD4^+^ effector cells and not iTregs. Mature DCs had a similar effect. Fortunately, semimature DCs [31] or DCs with a tolerogenic phenotype [32], such as the low expression of MHC or/and costimulatory molecules, had the ability to expand or generate Tregs de novo and promote the apoptotic death of effector T cells in some studies. Tolerogenic DCs were the optimal candidate to amplify the Foxp3^+^ iTregs, particularly in a mixed population of cells.

DCs have the unique ability to activate or suppress immune responses depending on their maturation status, phenotype, and tissue of origin. Due to their inherent plasticity, DCs are regarded as key instigators or regulators of innate and adaptive immunity. It is commonly held that conventional DCs have the unique ability to activate or suppress immune responses depending on their maturation status, whereas tolerogenic DCs exhibit the distinct functional properties in promoting tolerance. tDCs describe a broad range of immunoregulatory DCs that are usually immature [33], exhibit a plasmacytoid morphology [34], or are alternatively activated [15]. And on the basis of traditional definition, Jiang et al. [35] further hierarchically arranged two classes of maturation programs for DCs. They found that DCs matured by E-cadherin contained the phenotypic hallmarks of conventional mature DCs but without the secretion of inflammatory cytokines. And these E-cadherin-matured DCs could induce peripheral T cell tolerance in vivo and protect against EAE. Thus, alterations in adhesion could be seen as producing “phenotypically mature” immunogenic DCs, whereas microbial stimuli yield “functionally mature” immunogenic DCs under conditions of inflammation. Also, Vander Lugt et al. [36] provided a gene regulatory framework and indicated that the presence of DCs in peripheral tissues appeared phenotypically mature and had a critical role in peripheral tolerance, which illuminated the molecular underpinnings of DC maturation and function. Meanwhile, Guilliams et al. [37] provided a universal toolbox for the automated identification of DCs using unsupervised analysis of flow cytometry, paving the way toward the faithful identification of DC subsets to grasp the fascinating functional heterogeneity of DCs.

In this study, DCs exposed to IL-10 and TGF-*β* were resistant to maturation in response to LPS stimulation. The MFI of IA-IE, CD80, and CD86 expression on mtDCs was lower than the MFI on mDCs. Moreover, mtDCs secreted higher levels of IL-10 and TGF-*β* than mDCs, whereas IL-12 p40 production by mtDCs was negligible. Considering the limited cell expansion capability of tDCs, mtDCs matured by LPS were employed in this new method. mtDCs also displayed the tolerogenic surface phenotype and secreted a large number of “immunosuppressive” cytokines, whose tolerogenic characteristics were not significantly different from those of tDCs. More importantly, mtDCs effectively expand T cells, but tDCs always show a poor capacity to activate T cells. Due to their stable tolerogenic functions and improved ability to expand iTregs, mtDCs were employed as the best stimulator/inducer in this study. As a positive control, conventional mDCs were also added to expand iTregs in parallel experiments. As expected, fewer cells expanded by mDCs (iTreg_mDC_) expressed Foxp3 (37.4 ± 4.1%) than cells before amplification. Interestingly, these iTreg_mDC_ suppressed effector CD4^+^ T proliferation to a greater extent than iTregs in vitro, possibly because iTregs have enhanced functional activities after activation. Similarly, activated nTregs exert a stronger suppressive effect than silent nTregs [20]. This reason may be why iTreg_mtDC_, which are activated further by mtDCs, exerted more potent inhibitory effects than iTregs induced by TGF-*β* alone.

Noticeably, although more than 80% of the cells were Foxp3^+^ after the combined induction/expansion with TGF-*β* and mtDCs, approximately 10% CD4^+^ Foxp3^−^ cells and less than 5% CD11c^+^ mtDCs were observed. So iTreg_mtDC_ were the mixture of CD4^+^ Foxp3^+^ cells, CD4^+^ Foxp3^−^ cells, and mtDCs. Lan et al. compared the suppressive effects of a mixed population of iTregs and Foxp3^+^ iTregs and showed that these activated CD4^+^ Foxp3^−^ cells did not develop the ability to exert pathogenic effects after TGF-*β* treatment [10]. Thus, the purified Foxp3^+^ Treg population may not need to be sorted. Meanwhile, established murine arthritis is significantly inhibited by a tDC infusion [38, 39]. As shown in our previous study, mtDCs exerted suppressive effects in vitro and on the CIA mouse model after IL-10 and TGF-*β* polarization [13]. In this study, we did not isolate iTreg_mtDC_ from mtDCs; therefore, we cannot exclude the possibility that the very limited number of mtDCs infused with iTreg_mtDC_ may have contributed to the suppression of CIA.

Multiple mechanisms are involved in the inhibitory activities of iTregs in vivo. Our study has confirmed that different types of iTregs suppressed CIA in mice through similar mechanisms, including modulation of cytokine secretion, prolonged inhibition of anti-CII IgG antibodies, and polarization of the Treg/Th17 balance. Following treatment with iTreg_mtDC_, the serum levels of TNF-*α*, IL-17, and IL-6 were significantly reduced, whereas the levels of IFN-*γ*, IL-10, and TGF-*β* were markedly elevated in mice with CIA compared with the levels in mice treated with iTreg or iTreg_mDC_. In addition, iTreg_mtDC_ inhibit anti-CII-specific antibody responses more powerfully than iTregs or iTreg_mDC_, which contribute to the suppression of the development of CIA. Interestingly, the levels of IFN-*γ*, which is mainly produced by NK cells and Th1 cells and induces inflammation, were increased after the adoptive transfer of iTregs. In fact, an increasing number of studies have recently confirmed that IFN-*γ* acts as a disease-limiting factor in CIA and not a pathogenic factor [40, 41], though several studies have recently revealed a role for IFN-*α* in the pathogenesis of a subset of RA patients [42]. The protective mechanism of IFN-*γ* is to inhibit the differentiation of monocytes/macrophages into osteoclasts and suppress IL-1*β*-mediated MMP1 and MMP3 production by synovial fibroblasts in antigen-induced arthritis, thereby limiting cartilage degradation. More importantly, IFN-*γ* facilitates the development of Tregs from precursor T cells and increases their suppressive effects on CIA. Conversely, IFN-*γ* inhibits Th17 cell development and suppresses their effector functions. Therefore, the high level of IFN-*γ* may reduce inflammation and osteoclast differentiation in CIA mice. Additionally, another therapeutic benefit of iTregs is attributed to the fact that they express low levels of the IL-6 receptor and subsequent STAT-3 phosphorylation [43], express high levels of the Bcl-2 gene, and resist apoptosis [25], and, most importantly, iTregs shift the Treg/Th17 balance to a Treg-predominant phenotype [9]. Thus, a tolerant state was established following the iTreg treatment in vivo, which promoted Treg cell differentiation and proliferation while limiting the differentiation of Th17 cells in vivo. As expected, compared with iTregs or iTreg_mDC_, iTreg_mtDC_ showed more significant polarization of the Treg/Th17 balance in the CIA mice. Based on our in vitro experiments, iTreg_mtDC_ were the most effective cell type at reducing IL-17A secretion from CD4^+^ T cells and suppressing Th17 cell differentiation. Meanwhile, iTreg_mtDC_ more effectively inhibited antigen-specific CD4^+^ T cell expansion than iTregs at all tested S : R ratios, whereas a significant difference in the inhibition of antigen-specific CD4^+^ T cell expansion between iTreg_mtDC_ and iTreg_mDC_ was only observed at a low S : R ratio (1 : 8). Thus, iTreg_mtDC_ promoted a more potent tolerogenic microenvironment, a feature that was evidenced by the effectively reduced presentation of CIA symptoms and inhibition of CIA progression. Because iTreg_mtDC_ had the strongest antiarthritic effects, we were able to adoptively transfer low doses of iTreg_mtDC_ (1 × 10^6^ cells/CIA mouse) compared with the higher doses of iTregs (≥3 × 10^6^ cells/CIA mouse) required to achieve the same inhibition of established arthritis. Therefore, under the same culture condition, iTreg_mtDC_ meet the requirements for clinical application more readily than other iTregs, in terms of both the Foxp3^+^ cell purity and the number of cells produced.

In general, antigen-specific iTregs are believed to have a stronger suppressive ability than nonspecific iTregs [9]. However, in autoimmune diseases, such as RA and lupus, the specific antigens responsible for the disease processes remain undefined. Polyclonal iTreg_mtDC_ suppressed T cell proliferation and CIA, suggesting that manipulation of polyclonal iTreg_mtDC_ may represent a therapeutic tool with which to combat autoimmune diseases in which specific antigens have not been defined. Moreover, once the specific pathogenic antigens of RA are identified, mtDCs, potent APCs, could acquire and present specific antigens to iTregs, subsequently activating and propagating the antigen-specific iTregs. Thus, we believe that the involvement of mtDCs in the iTreg culture system will greatly improve future iTreg-induction/expansion methods.

In summary, we established a new iTreg polyclonal expansion method. Using this method, iTregs were efficiently expanded by tolerogenic DCs ex vivo to reach clinically relevant cell numbers while retaining and even strengthening their regulatory phenotypes and potent suppressor functions. In our study, these iTreg_mtDC_ prevented established CIA by modulating cytokine secretion, anti-CII antibodies, and the polarization of the Treg/Th17 balance. Based on these results, we confidently postulate that human mtDCs are a suitable stimulator to expand human Tregs to achieve clinically relevant cell numbers and enhance their inhibitory activity in vitro and in vivo, although some differences have been observed between human Tregs and mouse Tregs. Moreover, mtDCs are also potent APCs. mtDCs could acquire/present specific antigens to Tregs and then activate/propagate the antigen-specific Tregs, which are thought to have a stronger suppressive ability and better therapeutic effects than nonspecific iTregs. Thus, we have sufficient reasons to believe that the use of mtDCs in the iTreg culture system may contribute to improve future Treg-induction/expansion methods, establish a large-scale in vitro cell culture system for clinical applications, and enhance the therapeutic effects and extensive use of Treg-based cell therapy in the forthcoming decade.
